# Role of Magnetic Resonance Imaging in the Evaluation of Age- and Gender-Related Changes in the Dimensions of the Pituitary Gland in the Indian Population

**DOI:** 10.7759/cureus.54093

**Published:** 2024-02-12

**Authors:** Shikha Shajil, Praveen K Sharma, Aadithiyan Sekar, Govindarajan Rajendran, Aashika Amir

**Affiliations:** 1 Department of Radiology, Saveetha Medical College and Hospital, Saveetha Institute of Medical and Technical Sciences (SIMATS) Saveetha University, Chennai, IND

**Keywords:** longevity, analysis of variance, mental disorders, magnetic resonance imaging, pituitary diseases, pituitary gland

## Abstract

Background

MRI is the standard tool for imaging the pituitary gland. MRI is useful in detecting pathological conditions in the pituitary. Changes in the size and shape of the pituitary among different age groups are seen in MRI. Linear growth is seen in the pituitary during puberty except for growth spurts at the 1^st^, 10^th^, and 15^th^ years, followed by a decline in pituitary height and cross-sectional area with increasing age. A convex upper margin was seen in females more than in males. There is a shortage of information about pituitary dimensions and volume in various age groups and among both genders in the Indian population. Hence, a study is needed to assess these parameters.

Materials and methods

A retrospective cross-sectional study was done in the MRI unit of Radiology, Saveetha Medical College and Hospital, Chennai. A total of 200 patients in the age group of 11-80 years who underwent MRI free from neuroendocrine, neurological, and psychiatric disorders were included in this study.

Statistical analysis

Measurements were made of the pituitary gland's height, volume, and anteroposterior and transverse dimensions. Using SPSS Statistics software (IBM Corp. IBM SPSS Statistics for Windows. Armonk, NY: IBM Corp.), the data was input and examined. The ANOVA test revealed the relationship between anteroposterior dimension, transverse dimension, height, and volume with age. In contrast, an independent t-test determined the association of the same parameters with sex. The Chi-square test was used to assess the association of the shape of the pituitary gland with age and sex.

Results

Anteroposterior dimension, height, and volume of the pituitary gland were found to be statistically significant with age (p<0.05), but the transverse dimension was not significant with age (p>0.05). However, the independent t-test showed highly significant differences between the anteroposterior dimension in males and females. The shape of the pituitary gland was found to be statistically significant with age and gender. In contrast, the pituitary gland's transverse diameter, height, and volume showed no significance.

Conclusion

The study helps identify the substantial changes in the pituitary gland during a person's lifespan, which are affected by age and gender. The pituitary height and volume will reflect physiological neuroendocrine differences between younger and older male and female subjects.

## Introduction

Recent advances have made magnetic resonance imaging the method of choice for visualizing intracranial structures such as the sellar and parasellar regions [1]. MRI outperforms computed tomography and plain radiographs in investigating the sella, parasellar, and suprasellar regions. It differs from CT and conventional radiographs by providing high-resolution images [2].

Dynamic changes in the size and shape of the pituitary were documented while viewing cerebral structures on MRI in different age groups [3]. The size and shape of the pituitary gland are the most critical factors in diagnosing its pathology [4]. It includes physiological hypertrophy, empty sella, microadenoma, and inflammatory diseases. Standard pituitary gland measurements for the various age groups help diagnose borderline pituitary disorders.

The pituitary gland's size, shape, and volume reflect a change in the hormone physiology of the gland, depending on the age and gender of an individual [5]. The pituitary gland shows linear growth in puberty except for the 1^st^, 10^th^, and 15^th^ years. It undergoes physiological hypertrophy in puberty with increased pituitary size and a spherical or convex upper margin seen in girls, while boys change in pituitary size only [6].

Females aged 20-40 have larger pituitaries than males of the same age group. A convex upper pituitary margin is more common in younger females than in older females or males of any age. There is a decline in pituitary height and cross-sectional area with an increase in age, and it is more present in females than males [5]. A larger pituitary gland volume is present in females than in males [7]. The height of the pituitary gland varies with age: 6 mm for children under 12 years old (upper surface flat or slightly concave); 10 mm for puberty (upper surface convex; more in females); 8 mm for males, 9 mm for females, and 12 mm for pregnant young adults [4].

The need for the study arises from the lack of measurement of pituitary dimensions and volume in various age groups and among both genders in the Indian population. Due to the shortage of such information, a study was conducted to assess the role of magnetic resonance imaging in evaluating age- and gender-related changes in dimensions of the pituitary gland in the Indian population.

## Materials and methods

The retrospective cross-sectional study was conducted at the MRI unit within the Department of Radiology at Saveetha Medical College and Hospital in Chennai. The study aimed to analyze brain MRI data from a cohort of 200 patients aged 11 to 80 years. All patients who were free from neuroendocrine, neurological, and psychiatric disorders were included in the study. Pregnant women, breastfeeding mothers, women on oral contraceptive pills, and patients with pituitary pathology were excluded. Patients with metallic implants and those with neurological disorders were also excluded from the study. Patients were categorized into six distinct age groups: 11-20 years, 21-30 years, 31-40 years, 41-50 years, and above 60 years. The study period spanned from January 1^st^, 2022, to January 30^th^, 2022. All MRI scans were performed using a Philips 1.5 Tesla Multiva MRI scanner (Philips, Amsterdam, Netherlands). Sagittal and coronal images were generated using spin echo imaging techniques. The sagittal scan protocol featured a matrix of 240 x 240 with a field of view (FOV) of 240 mm and a 1 mm isometric voxel. For the coronal section, the protocol included a matrix of 324 x 324, a FOV of 233 mm, and a 5 mm slice thickness. Measurements of the pituitary gland were taken from the mid-sagittal T1 image for the anteroposterior dimension (a), the craniocaudal dimension or height (h), and the coronal image for the transverse size (t). The pituitary gland shape was classified as flat, concave, or convex, and its volume was calculated using the formula Volume = 0.52 aht, with dimensions measured in millimeters and volume in cubic millimeters. Statistical analysis was performed using SPSS Statistics software (IBM Corp. IBM SPSS Statistics for Windows. Armonk, NY: IBM Corp.). The association of the anteroposterior dimension, transverse dimension, mean height, and volume with age was determined using the ANOVA test. Additionally, an independent t-test was utilized to assess the association of these parameters with sex. The shape of the pituitary gland was analyzed for associations with age and sex using the chi-square test. A significance level of p<0.05 was considered statistically significant. This study's design and methodology provide a comprehensive framework for analyzing pituitary gland dimensions and shape variations across different age groups and sexes. By employing rigorous imaging protocols and statistical analyses, the study aims to contribute valuable insights into the understanding of pituitary gland morphology in a diverse patient population.

## Results

**Figure 1:**
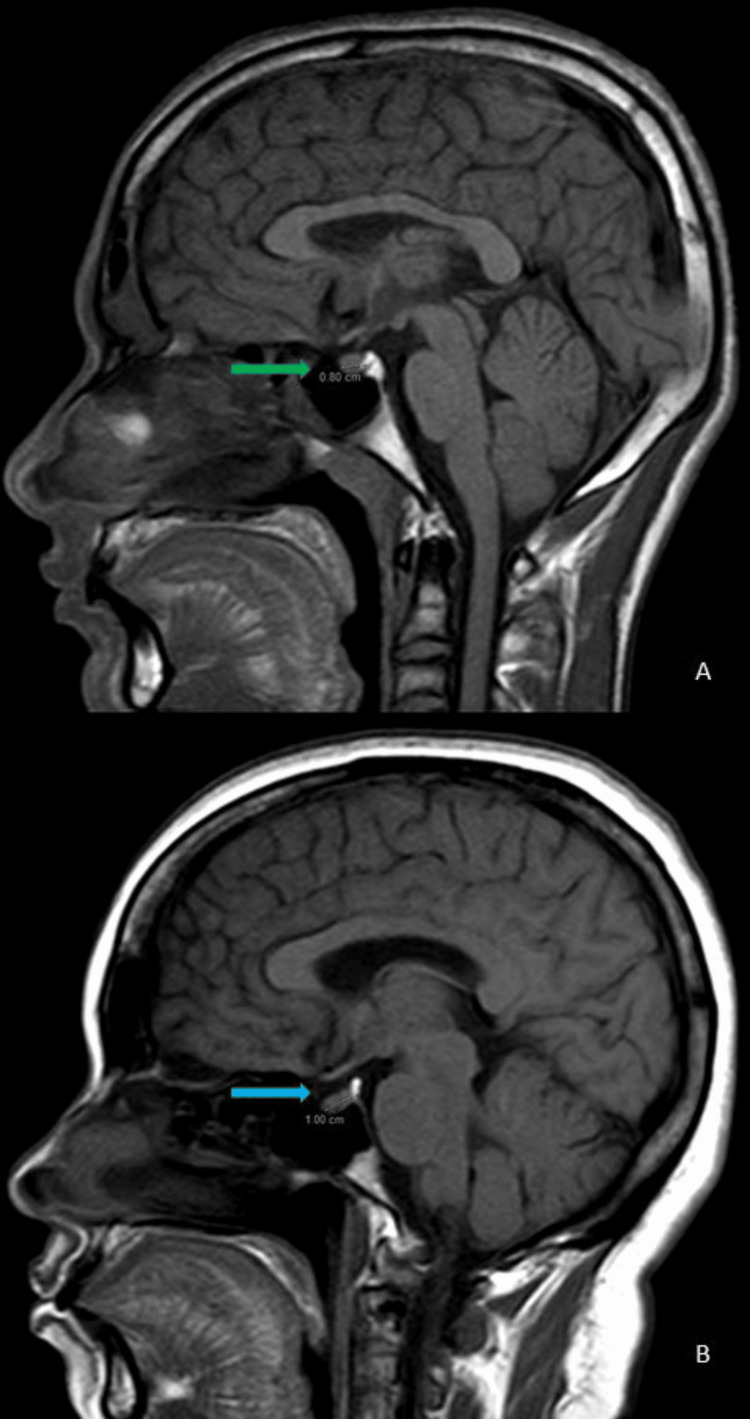
(A, B): MRI of the sella turcica. (A) T1 mid-sagittal: the pituitary gland in a 45-year-old female shows an anteroposterior dimension of 8 mm (green arrow). (B) T1 mid-sagittal: the pituitary gland in a 25-year-old male shows an anteroposterior dimension of 10 mm (blue arrow)

The MRI images of 200 patients were examined, of which 104 (52%) were males and 96 (48%) were females, with the ages of the patients varying from 11 to 80 years. The mean anteroposterior dimension of the pituitary in the study group was 9.56 + 1.38 mm, with the mean transverse size, mean pituitary height, and volume being 11.95 + 1.82 mm, 5.39 + 1.21 mm, and 322.05 + 104.96 mm3, respectively. On observing the MRI images, the most common shape of the pituitary was found to be flat (42%), followed by convex (30%), and then concave (28%). The maximum mean value of the anteroposterior dimension was found in the age group of 21-30 years, and the least was in the age group of 41-50 years (Figure 1).

**Figure 2:**
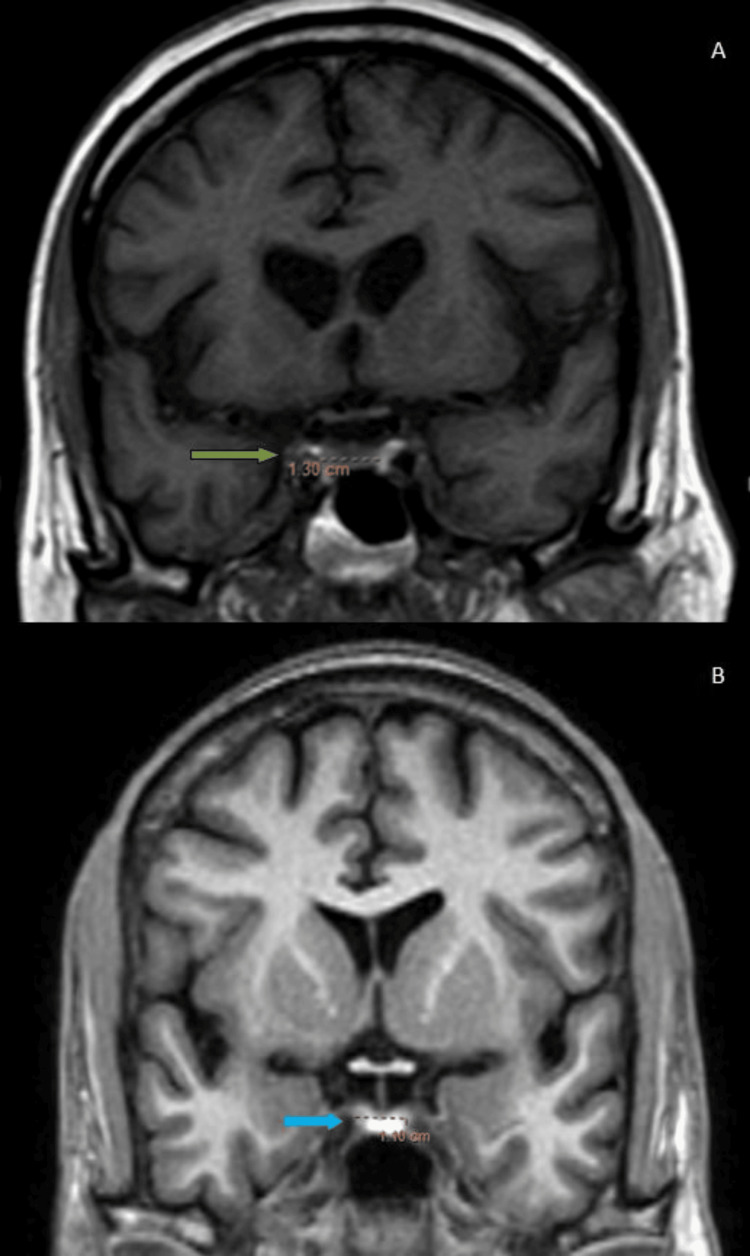
(A, B): MRI of the sella turcica. (A) T1 mid-coronal: the pituitary gland in a 35-year-old male shows a transverse dimension of 13 mm (green arrow). (B) T1 mid-coronal: the pituitary gland in a 45-year-old female shows a transverse dimension of 11 mm (blue arrow)

For the transverse dimension, the maximum mean value was found in the age group 31-40 years, and the minimum mean value was found in 41-50 years (Figure 2). The mean pituitary height and mean volume were maximum in the 11-20 age group and minimum in patients >60. Anteroposterior dimension, height, and volume of the pituitary gland were statistically significant with age (p<0.05). Still, the transverse dimension was insignificant with age (p>0.05) (Table 1).

**Table 1:** Mean anteroposterior dimension, transverse, height, and volume of the pituitary gland according to age

Age (in years)	Number	Anteroposterior (in mm)	Transverse (in mm)	Height (in mm)	Volume (in mm^3^)
		Mean+SD	Mean+SD	Mean+SD	Mean+SD
11-20	25	9.12 + 0.63	12.20 + 2.13	6.52 + 1.23	378.75 + 108.27
21-30	36	10.7 + 1.64	12.19 + 1.84	5.28 + 0.99	357.99 + 104.25
31-40	50	9.38 + 1.39	12.23 + 2.08	5.27 + 1.11	318.76 + 112.13
41-50	41	8.97 + 0.97	11.30 + 1.85	5.30 + 1.27	280.79 + 94.50
51-60	26	9.88 + 1.28	12.09 + 0.96	5.24 + 0.99	327.20 + 87.92
>60	22	9.34 + 1.21	11.70 + 1.29	4.91 + 1.27	277.11 + 76.15
F-value	200	8.953	1.632	5.865	4.821
p-value	200	0.000	0.153	0.000	0.000

Mean anteroposterior dimensions, mean transverse dimensions, and pituitary gland volumes were higher in males than females. Nonetheless, it was discovered that women had a greater mean height than men. Independent t-tests showed highly significant differences between the anteroposterior dimension in males and females. In contrast, the pituitary gland's transverse size, height, and volume showed no significance (Table 2).

**Table 2:** Mean anteroposterior dimension, transverse, height, and volume of the pituitary gland according to gender

Gender	Number	Anteroposterior (in mm)	Transverse (in mm)	Height (in mm)	Volume (in mm^3^)
		Mean + SD	Mean + SD	Mean + SD	Mean + SD
Male	104	9.85 + 1.43	12.06 + 1.91	5.35 + 1.18	332.63 + 107.64
Female	96	9.25 + 1.26	11.83 + 1.73	5.43 + 1.25	310.60 + 101.30
t-value		3.141	0.871	-0.426	1.487
p-value		0.001	0.384	0.669	0.138

**Figure 3:**
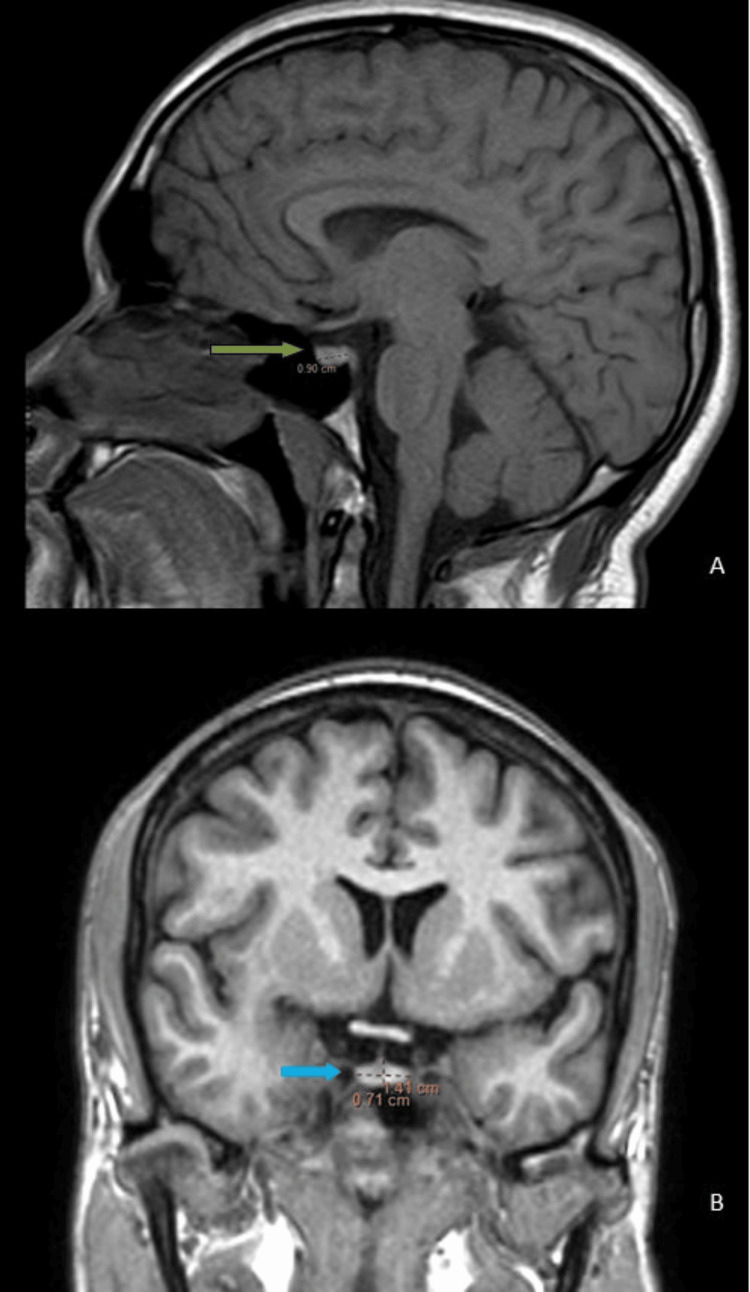
(A, B): MRI of the sella turcica. (A) T1 mid-sagittal: the pituitary gland in a 17-year-old male shows an anteroposterior of 9 mm (green arrow). (B) T1 mid-coronal: the pituitary gland of the same male shows a transverse dimension and height of 14 mm and 7 mm, respectively (blue arrow). The pituitary gland volume is 441 mm3

**Figure 4:**
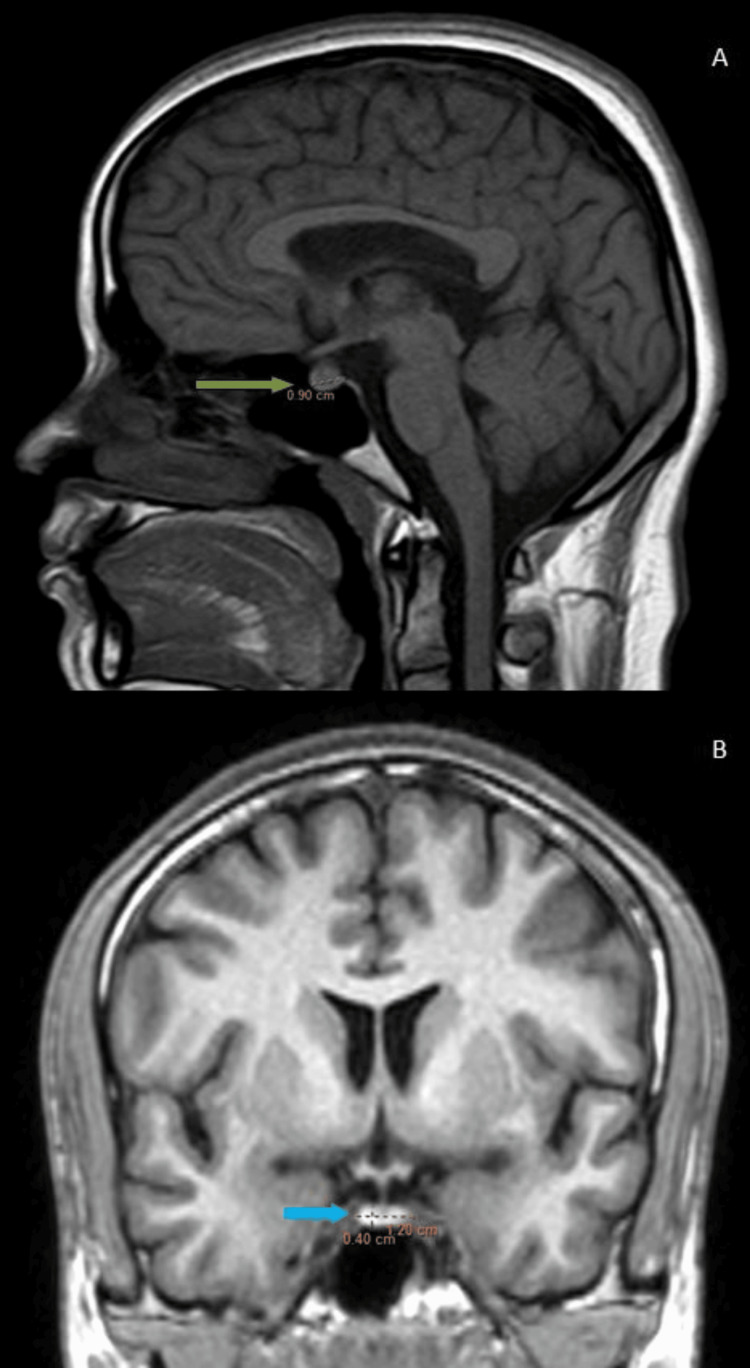
(A, B): MRI of the sella turcica. (A) T1 mid-sagittal: the pituitary gland in a 50-year-old male shows an anteroposterior of 9 mm (green arrow). (B) T1 mid-coronal: the pituitary gland of the same patient shows a transverse dimension and height of 12 mm and 5 mm, respectively (blue arrow). The pituitary gland volume is 270 mm3

**Figure 5:**
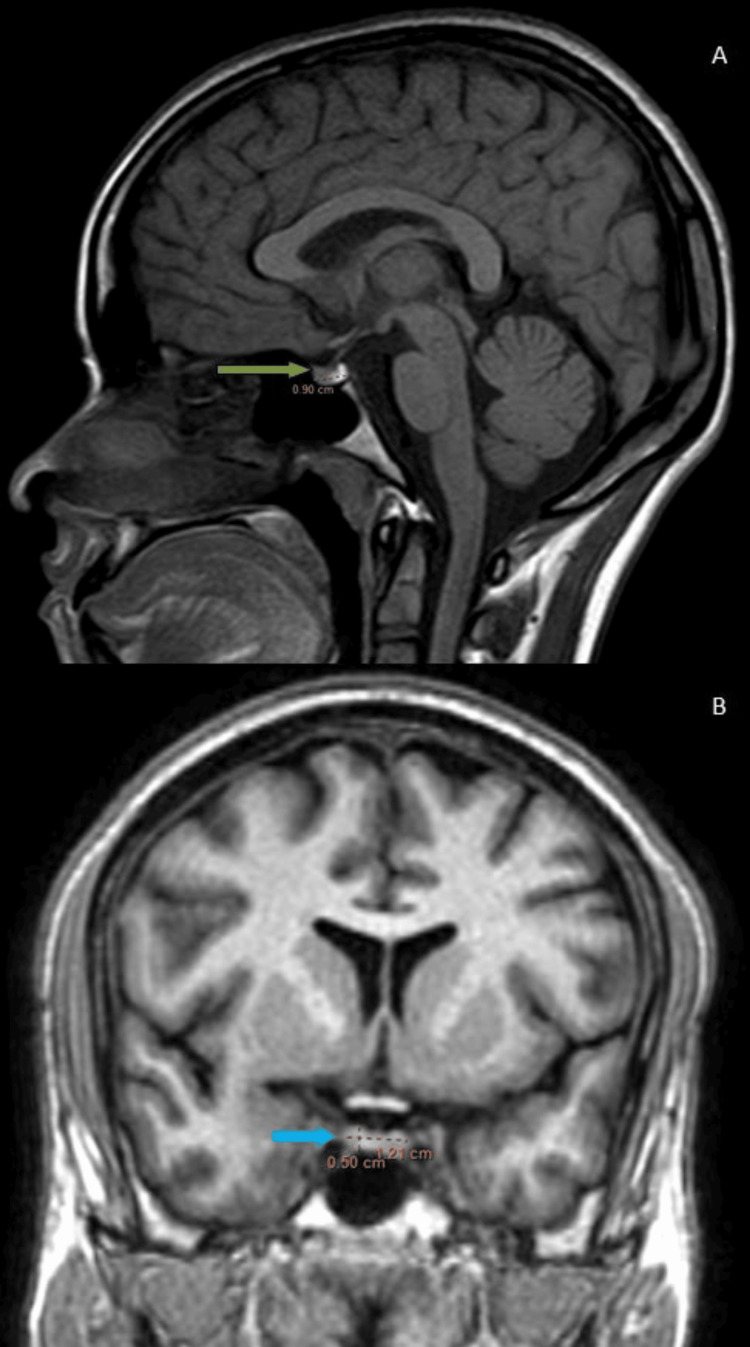
(A, B): MRI of the sella turcica. (A) T1 mid-sagittal: the pituitary gland in a 65-year-old female shows an anteroposterior of 9 mm (green arrow). (B) FLAIR mid-coronal: the pituitary gland of the same female shows a transverse dimension and height of 12 mm and 4 mm, respectively (blue arrow). The pituitary gland volume is 216 mm3

Maximum pituitary height and volume were observed in the age group of 11-20 years for both males and females (Figure 3, Table 3). Male patients aged 41 to 50 had the lowest pituitary volume (Figure 4, Table 3), whereas those older than 60 had the lowest pituitary height. Female patients older than 60 had the lowest pituitary height and volume (Figure 5, Table 3).

**Table 3:** Mean pituitary volume and pituitary height in male and female gender across different age groups

Age group (years)	Gender	Mean volume (in mm^3^)	Mean height (in mm)
11-20	Male	384.40 + 80.61	6.46 + 1.20
	Female	373.54 + 132.02	6.56 + 1.31
21-30	Male	353.50 + 110.50	5.04 + 0.98
	Female	361.20 + 102.21	5.46 + 0.97
31-40	Male	341.32 + 126.80	5.31 + 0.99
	Female	284.92 + 76.66	5.20 + 1.30
41-50	Male	281.06 + 90.95	5.16 + 1.08
	Female	280.50 + 100.46	5.45 + 1.46
51-60	Male	357.30 + 101.61	5.54 + 1.23
	Female	297.11 + 61.79	4.94 + 0.58
Above 60	Male	299.33 + 79.71	4.93 + 1.44
	Female	245.01 + 61.07	4.90 + 1.05

**Figure 6:**
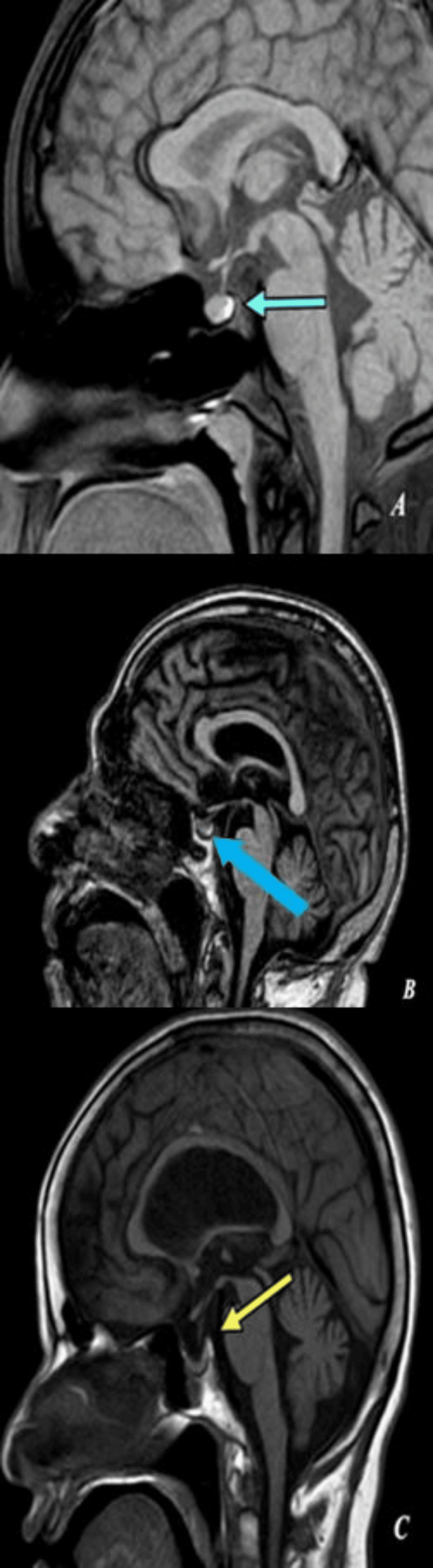
(A, B, C): MRI of the sella turcica. (A) T1 mid-sagittal: the pituitary gland in a 20-year-old male shows that the upper border is convex in shape (green arrow). (B) T1 mid-sagittal: the pituitary gland in a 62-year-old male shows that the upper border is flat in shape (blue arrow). (C) T1 mid-sagittal: the pituitary gland in a 55-year-old female shows that the upper border is concave in shape (yellow arrow)

**Figure 7:**
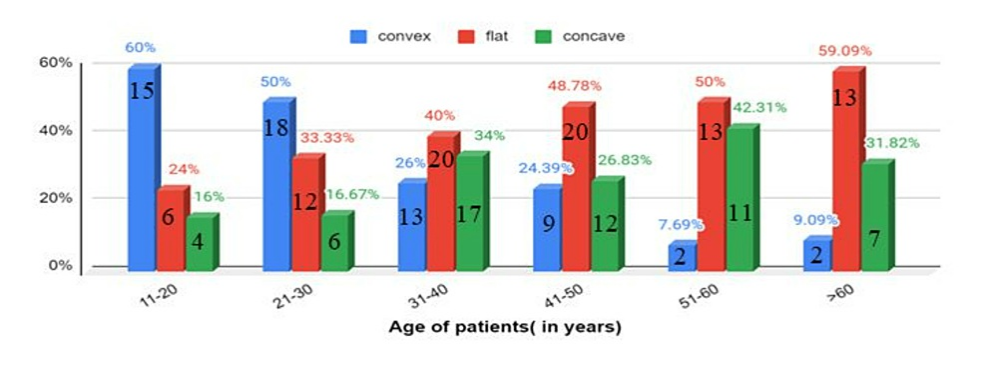
Shape of the pituitary gland among different age groups

The convex upper surface of the pituitary gland was most commonly observed in age groups 11-20 years (60%) and 20-30 years (50%). After that, its incidence decreased with an increase in age. However, the presence of flat and concave shapes increased as age progressed. The flat shape of the pituitary was found to be highest in the age group >60 years, while the incidence of concave shape was maximum in the age group 51-60 years (Figures 6-7).

**Figure 8:**
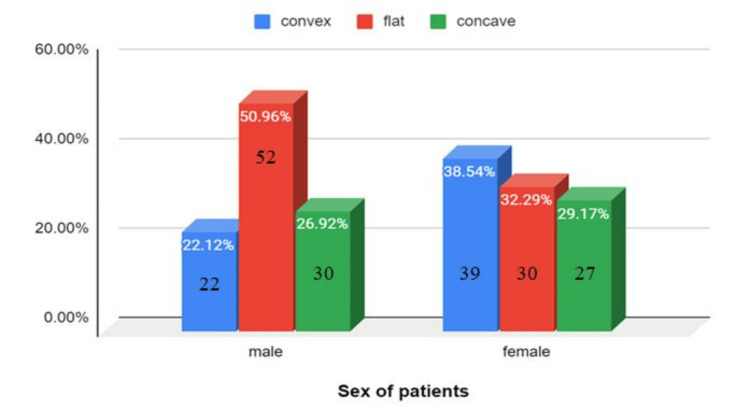
Shape of the pituitary gland among males and females

In males, flat surfaces were most common (50.96%), followed by concave (26.92%), and then convex (22.12%). In females, the most common shape was convex (38.54%), followed by flat (32.29%), and then concave (29.17%) (Figure 8). The shape of the pituitary gland was found to be statistically significant with age and gender (p<0.05).

## Discussion

The study was carried out in the MRI unit of Radiology at Saveetha Medical College and Hospital, Chennai. In this study, 104 (52%) were males compared to 96 (48%) females, similar to the survey done by Najeeb et al., which had 112 males (55.17%) and 91 females (44.83%) [8]. Most patients whose MRI scans were examined were in the age group of 31-40 years, similar to the study by Khanal et al. [5]. At the same time, most studies focus on height, such as the study by Tsunoda et al. [9]. Our analysis considers all the parameters, such as anteroposterior dimension, height, and the transverse dimension of the pituitary, as the size and shape of a normal pituitary gland can vary according to age, gender, and the hormonal environment of the patient. Pituitary height is at its maximum in the age group of 11-20 years, with females having higher values than males, followed by a gradual reduction in height with increasing age. Yadav et al. did a similar study [10]. During puberty, height increases due to increased luteinizing hormone production and differences in physiology in neuroendocrine hormones in younger and older patients. The decrease in pituitary height with age is caused by changes in endocrine status as well as physiological atrophy of the gland [11]. Pituitary height was statistically significant with age, which correlates to the research done by Maskey et al. [12]. The results obtained in this study demonstrated a gradual linear increase in pituitary volume over the first thirty years of life, consistent with the study by Mangieri et al. [13].

Pituitary volume was found to be statistically significant among different age groups, similar to the study done by Kumar [14]. The maximum anteroposterior dimension was seen in 20-29 years, which aligns with the survey by Sanjay et al. [15]. Pituitary height, volume, and transverse dimension were not statistically significant with sex. Still, pituitary volume was more prominent in males than females, similar to the study by Ibinaiye et al. [16].

The anteroposterior dimension showed statistical significance with sex, which follows the study by Maskey et al. [12]. When examining the MRI images, flat (4%) shapes were the most prevalent, followed by convex (30%) and concave (28%). This correlates with the study by Najeeb et al. [8], which stated flat was most common (46.8%), followed by convex (31.03%) and concave (20.7%).

The incidence of the upper surface of the pituitary gland being convex was highest in the age groups 11-20 years (60%) and 21-30 years (50%), gradually decreasing as age progressed. However, most participants showed an increase in the incidence of flat and concave shapes of the pituitary as age progressed. The most common shape found in males was flat (50.96%), followed by concave (26.92%) and convex (22.12%), while the most common shape in females was convex (38.54%), followed by flat (32.29%) and concave (29.17%). These findings coincide with the study done in Nepal [4]. The major limitations of our study were the single-center design, which limited the generalizability of the findings to other ethnic groups, and the retrospective nature of the study.

## Conclusions

This study uses an MRI scan to provide data regarding the standard dimensions and shape of the pituitary gland across various age groups and genders. The study helps us identify substantial changes in the pituitary gland during a person's lifespan, dependent on age and gender. The pituitary height and volume will reflect physiological neuroendocrine differences. That occurs in all ages and genders (younger, older males, and females). Any abnormal variation in the pituitary gland's dimensions and size can aid in identifying any pathology and help form an early diagnosis. Hence, MRI can be a valuable tool to determine the pituitary size accurately, correlating the findings with age and sex, and determining any pathology.
